# Interaction and Entanglement of a Pair of Quantum Emitters near a Nanoparticle: Analysis beyond Electric-Dipole Approximation

**DOI:** 10.3390/e22020135

**Published:** 2020-01-23

**Authors:** Miriam Kosik, Karolina Słowik

**Affiliations:** Institute of Physics, Faculty of Physics, Astronomy and Informatics, Nicolaus Copernicus University in Toruń, Grudziadzka 5, 87-100 Torun, Poland

**Keywords:** quantum plasmonics, beyond dipole, entanglement

## Abstract

In this paper, we study the collective effects which appear as a pair of quantum emitters is positioned in close vicinity to a plasmonic nanoparticle. These effects include multipole–multipole interaction and collective decay, the strengths and rates of which are modified by the presence of the nanoparticle. As a result, entanglement is generated between the quantum emitters, which survives in the stationary state. To evaluate these effects, we exploit the Green’s tensor-based quantization scheme in the Markovian limit, taking into account the corrections from light–matter coupling channels higher than the electric dipole. We find these higher-order channels to significantly influence the collective rates and degree of entanglement, and in particular, to qualitatively influence their spatial profiles. Our findings indicate that, apart from quantitatively modifying the results, the higher-order interaction channels may introduce asymmetry into the spatial distribution of the collective response.

## 1. Introduction

When subject to resonant illumination, plasmonic nanoparticles are able to focus electromagnetic fields to subwavelength volumes of space [1,2,3]. Such a tight field confinement is accompanied by a corresponding local field intensity enhancement of up to three orders of magnitude [2]. In quantum plasmonics [4], this effect is usually exploited to boost the interaction strengths between the locally enhanced light and quantum emitters positioned in the hotspots near the nanoparticles. Typically, these quantum emitters are molecules, quantum dots, or crystalline defects. The achieved interaction strengths typically reach the THz regime [5,6], but can be of the order of electron volt [7], outperforming even photonic crystal cavities [8].

These remarkable interaction strengths enable the fast addressing of quantum emitters with light: even in the weak-coupling regime, a quantum transition can occur at timescales of picoseconds, while stationary states are reached within nanoseconds [5,9]. This effect is typically studied in terms of Purcell factors [10], which quantifies the enhancement of the spontaneous emission rate of quantum emitters due to neighboring nanoparticles [11,12].

In this work, we adopt the quantum-optical perspective, according to which a spontaneous emission is a result of a purely quantum origin, arising from a coupling of a quantum emitter to a surrounding electromagnetic field in its vacuum state [13]. The enhancement of a spontaneous emission, however, can be calculated through classical means, as the power enhancement of a source represented by a classical electric dipole, magnetic dipole, or another type of source. The enhancement is conveniently expressed in terms of electromagnetic Green’s tensor [14,15]. If multiple quantum emitters are present in the close vicinity of a nanoparticle, they may all couple to the surrounding quantum vacuum. As a result, additional interesting phenomena arise. In particular, the quantum vacuum surrounding the nanoparticle can serve as a carrier for interactions between the emitters [16]; plasmon-enhanced dipole–dipole coupling was studied in [17,18,19]. This result is derived from the field elimination from the description either in a formalism based on adiabatic elimination of leaky electromagnetic modes [20], or more rigorously, using the electromagnetic Green’s tensor-based field quantization in dispersive media and the Markovian approximation [14,19,21,22,23].

The confinement to subwavelength spatial domains implies that the assumptions of the paradigmatic electric-dipole approximation may break, and higher-order multipolar channels of interaction between matter and light should be taken into account. This is because the electric-dipole approximation is valid if the size-scale of the field modulations is significantly larger than the extent of the quantum emitter, which may not be the case near plasmonic nanoparticles. The significance of higher-order multipolar terms has been suggested [24,25,26,27,28,29] and verified experimentally [30,31,32,33,34]. Their impact is not only quantitative: the presence of several parallel interaction channels, for example electric and magnetic dipolar or electric quadrupolar, unlocks the possibility of interference [35]. The appealing consequence of destructive interference is that it might lead to spontaneous emission lifetimes enhanced with respect to the free-space values, corresponding to a perspective of linewidths reduced below the “natural level”. In the context of realization of quantum information protocols, the increased lifetime might enable quantum information storage in the quantum emitter’s excited state for longer times, which might be realized in nanoscale platforms. These effects can be evaluated based on the theory developed in [36].

Naturally, multiple emitters near a nanoparticle could be used to store not only a single excitation per emitter encoding a single quantum bit, but also correlations in the form of quantum entanglement. This effect has been studied before in the plasmonic context [18,20] within the electric-dipole approximation, and has been suggested for the generation of squeezed light [37].

Here, we study how higher-order interaction terms might influence the collective properties of a pair of emitters positioned near a spherical nanoparticle, which belongs to the most typical of geometries investigated in theory and experiments. The studied scenario involves external illumination with a plane wave drive, and the collective properties include the effective inter-emitter coupling, decay rate, and degree of entanglement. We confirm the important impact of higher-order light–matter interaction channels of both a quantitative and a qualitative character.

## 2. Results

In this section, we introduce the investigated system (Section 2.1) and briefly recapitulate on the theory developed to a large extent in our previous work [36], though extended by the inclusion of classical illumination and studies of entanglement of quantum emitters (Section 2.2). We perform a study of the collective phenomena beyond the electric-dipole channel with an example in the third part of Section 2.3.

### 2.1. System

The investigated system consists of a pair of two-level quantum emitters, with excited states ${|e\rangle }_{j}$ and ground states ${|g\rangle }_{j}$, where j∈{1,2} gives the numbers of the emitter. The eigenstates are separated by energy differences $\hbar \omega_{j}$, where *ℏ* stands for the reduced Planck constant. Each quantum emitter is described by the set of Pauli operators ${\{\sigma_{j}=|g\rangle }_{j}\langle e{|}_{j},\;\sigma_{j}^{†}\}$. We introduce their extensions to the Hilbert space of the pair of emitters $\Sigma_{1}=\sigma_{1}⊗1\;\;1$, $\Sigma_{2}=1\;\;1⊗\sigma_{2}$, where 11 is an identity operator in the Hilbert space of a given quantum emitter. Our goal is to investigate the stationary entanglement of such a pair of emitters located near a plasmonic nanoparticle whose exemplary geometry is described below. In such a nanoscale setup, a realistic scenario to generate entanglement involves the illumination of the system with an external laser beam. Then, the quantum emitters are coupled to the electromagnetic field of the following electric Fourier components:

(1) $$E\left(r,\omega \right)=\sum_{X=C,V}E_{\mathrm{inc},X}\left(r,\omega \right)+E_{\mathrm{scat},X}\left(r,\omega \right).$$

The subscript “inc” stands for the incoming field, which is the illumination which combines a weak laser beam approximated as a classical plane wave (subscript “C” for classical), $E_{\mathrm{inc},C}\left(r,\omega \right)=E_{\mathrm{drive}}\left(r,\omega_{\mathrm{drive}}\right)\delta \left(\omega -\omega_{\mathrm{drive}}\right)+E_{\mathrm{drive}}^{★}\left(r,\omega_{\mathrm{drive}}\right)\delta \left(\omega +\omega_{\mathrm{drive}}\right)$, and the background of quantum vacuum fluctuations

(2) $$E_{\mathrm{inc},V}\left(r,\omega \right)=i\mu_{0}\omega \int d^{3}r^{\prime }G_{0}\left(r,r^{\prime },\omega \right)j_{V}\left(r^{\prime },\omega \right),$$

where $\mu_{0}$ is the vacuum magnetic permeability and $G_{0}\left(r,r^{\prime },\omega \right)$ is the electromagnetic Green’s tensor of a homogeneous medium connecting the source at a position $r^{\prime }$ to the field at a position r.

The subscript “scat” in Equation (1) represents the field scattered at the nanoparticle given by

(3) $$E_{\mathrm{scat},X}\left(r,\omega \right)=i\mu_{0}\omega \int d^{3}r^{\prime }G_{\mathrm{scat}}\left(r,r^{\prime },\omega \right)j_{X}\left(r^{\prime },\omega \right),$$

where $G_{\mathrm{scat}}\left(r,r^{\prime },\omega \right)$ is the electromagnetic Green’s tensor representing the scattered part of the electromagnetic field. The source is a current density induced in the nanoparticle either by the classical plane wave ($j_{C}\left(r^{\prime },\omega \right)$) or by the vacuum noise ($j_{V}\left(r^{\prime },\omega \right)$). Naturally, the electric part of the field is accompanied by the magnetic one $B\left(r,\omega \right)=-\frac{i}{\omega }\nabla \times E\left(r,\omega \right),$ where *i* is the imaginary unit. The magnetic field can be decomposed into the incoming and scattered, classical and vacuum-induced components, accordingly.

In general, both the electric and the magnetic components of the field can be coupled to the quantum emitters, i.e., to the electric dipole $d_{j}$, magnetic dipole $m_{j}$, electric quadrupole $Q_{j}$, and higher-order multipolar moments characterizing the quantum transition between the eigenstates. These transition moments are expressed through the matrix elements of the corresponding operators $d_{j}=\langle e|{\hat{d}}_{j}|g\rangle$, and similarly for the other multipoles. In this work, we assume the emitters do not support permanent multipolar moments. For more details on multipolar coupling, please see [33,36,38]. Since we work far from the ultrastrong coupling regime, we assume the rotating wave approximation to hold, and apply it in the following interaction Hamiltonian taking into account the electric dipole, magnetic dipole, and electric-quadrupole terms [38]:

(4) $$H_{\mathrm{int}}=-\sum_{j}\left\{\left[E^{-}\left(r_{j}\right)\cdot d_{j}+B^{-}\left(r_{j}\right)\cdot m_{j}+\nabla E^{-}\left(r_{j}\right):Q_{j}\right]\Sigma_{j}+\Sigma_{j}^{†}\left[d_{j}^{†}\cdot E^{+}\left(r_{j}\right)+m_{j}^{†}\cdot B^{+}\left(r_{j}\right)+Q_{j}^{†}:\nabla E^{+}\left(r_{j}\right)\right]\right\},$$

where in the Schrödinger picture $E^{-}\left(r\right)=\int_{0}^{\infty }d\omega E\left(r,\omega \right)$, $E^{+}\left(r\right)={\left[E^{-}\left(r\right)\right]}^{†}$, and $E\left(r\right)=E^{-}\left(r\right)+E^{+}\left(r\right)$. The fields are evaluated at the quantum emitters’ positions $r_{j}$. In the expression above, the dot · denotes a scalar product, ∇E is a dyadic product, and $C:D=\sum_{ij}C_{ij}D_{ji}$ is a double-dot product of tensors *C* and *D*.

We would like to now discuss the roles played by different field components in the scenario proposed in this work. Both components, the quantum vacuum and the classical drive, enter the Hamiltonian above.

- The quantum vacuum is a background, playing the role of a carrier of the interactions of the quantum emitters. In open systems like the one considered in this work, this part of the field is tricky to keep track of, since it involves a continuum of optical modes. However, in this case, the quantum vacuum surrounding the emitters can be treated as a reservoir shared between the quantum emitters [16]. Then, it can be eliminated from the evolution equations of the system, leading to an effective picture as given below. The effective picture is obtained in the Markovian approximation, based on the assumption (which is well met in most practical cases) that at considered timescales, the light–matter coupling introduces only a small perturbation to the free dynamics of the field and of the emitter (for rigorous derivations and detailed discussions please see [23,36]). Under the Markovian approximation, one arrives at an effective form of equations describing the evolution of the emitters alone, in which contributions from Lamb shifts $\delta_{j}$ and spontaneous emission rates can be distinguished for individual emitters, and additionally, direct multipole–multipole interactions and collective decay effects arise between multiple emitters. These effects already arise in free space, but can be significantly enhanced and modified in the presence of plasmonic nanoparticles tailoring the properties of the quantum vacuum, quantified with the Green’s tensor. Please note that the coupling effect holds even if the frequencies of the emitters are not identical, as long as their difference $|\omega_{1}-\omega_{2}|$ is much smaller than the width of the plasmonic resonance;
- The classical drive is the source of energy in the setup and therefore is necessary to enable stationary entanglement generation. The energy it provides trades off various decay channels described below. Without the drive, the stationary state of the system would be the ground state, which is separable.

### 2.2. Hamiltonian and Liouvillan

As described in detail in [36], under the Markovian approximation, Equation (4) reduces to the following form of the full Hamiltonian, given here in a frame rotating with the frequency of the driving field $\omega_{\mathrm{drive}}$:

(5) $$H/\hbar =\sum_{j=1,2}\left(\delta \omega_{j}\Sigma_{j}^{†}\Sigma_{j}+\Omega_{j}\Sigma_{j}^{†}+\Omega_{j}^{★}\Sigma_{j}\right)+\xi \Sigma_{2}^{†}\Sigma_{1}+\xi^{★}\Sigma_{1}\Sigma_{2}^{†}.$$

Here, $\delta \omega_{j}=\omega_{j}+\delta_{j}-\omega_{\mathrm{drive}}$ is the detuning of the drive from the transition frequency of the jth system corrected by the effective Lamb shift. It corresponds to an effective energy shift of the *j*th emitter. The effective coupling strengths with the classical field are

(6) $$\hbar \Omega_{j}=-E_{C}\left(r_{j},\omega_{\mathrm{drive}}\right)\cdot d_{j}-B_{C}\left(r_{j},\omega_{\mathrm{drive}}\right)\cdot m_{j}-\left[\nabla E_{C}\left(r_{j},\omega_{\mathrm{drive}}\right)\right]:Q_{j}.$$

They describe interactions with the classical part of the field consisting of the illuminating plane wave and the part scattered at the nanoparticle $E_{C}\left(r\right)=\int_{0}^{\infty }d\omega \left[E_{\mathrm{inc}},C\left(r,\omega \right)+E_{\mathrm{scat}},C\left(r,\omega \right)\right]$ and similarly for the magnetic field.

The final contribution to the Hamiltonian describes the first among the collective effects, i.e., the multipole–multipole interaction of strength ξ. This quantity arises from the presence of the quantum vacuum and is essential for the purpose of entanglement generation since it is the very source of nonclassical correlations of the two emitters. We consider the coupling strength in the form extended with respect to the well-known one corresponding to the electric-dipole approximation (see [17,19] for a result in the electric-dipole approximation and [36] for extensions).

(7) ξ=πω¯2∑mnRmnω¯ReGmnr′,r,ω¯|r=r1,r′=r2+Imnω¯ImGmnr′,r,ω¯|r=r1,r′=r2,

where $\bar{\omega }=\frac{1}{2}\left(\omega_{1}+\omega_{2}\right)$ and $R_{mn},I_{mn}$ correspond to the real and imaginary parts of a differential operator acting on the elements of the full Green’s tensor $G_{mn}={\left(G_{0}+G_{\mathrm{scat}}\right)}_{mn}$,

(8) Rmn(ω)=μ0πℏReDj,mr′†ωDj,nrω,Imn(ω)=μ0πℏImDj,mr′†ωDj,nrω,

with components of the differential operator

(9) $$D_{j,n}^{r}\left(\omega \right)=d_{j,n}+\sum_{k}\left(Q_{j,nk}+\frac{i}{\omega }\sum_{p}\epsilon_{pkn}m_{j,p}\right)\frac{\partial }{\partial r_{k}},$$

and with n,k,p∈{x,y,z}. Here, $d_{j,n}$ stands for the *n*th spatial component of the transition dipole moment element of the jth quantum emitter, and similarly for the other multipoles. From the structure of Equations (7) and (9), it is clear that the interaction will contain two sorts of terms, i.e., of “pure” and “mixed” origin. To explain their meaning, we consider each quantum emitter as a complicated source combining the electric-dipole, magnetic-dipole, and electric-quadrupole components. Each of these multipolar components is a source of electric and magnetic fields, which are scattered at the nanoparticle. As the other emitter interacts with these scattered fields, we can distinguish

- “Pure” coupling channels, in which the electric-dipole moment of the emitter j interacts with the electric field originating from the electric-dipole moment of the emitter $j^{\prime }$, the magnetic-dipole moment of the emitter j is coupled to the magnetic field generated by the magnetic-dipole moment of the emitter $j^{\prime }$, and the electric-quadrupole moment of the emitter j- to the modulations of the electric field originating from the electric-quadrupole source corresponding to the emitter $j^{\prime }$;
- “Mixed” coupling channels related to interference, for example, an electric-dipole moment of the jth emitter coupled to the electric field generated by the magnetic dipole or electric-quadrupole sources related to the emitter $j^{\prime }$, etc.

The same sorts of channels will be distinguished in collective decay and will influence the degree of entanglement.

Please note that in the electric-dipole approximation, the operator in Equation (9) reduces to an element of the electric-dipole moment, and in free space, the expression for ξ reduces to the familiar form $\hbar \xi =\frac{1}{4\pi \epsilon_{0}}\frac{d_{j}\cdot d_{j}^{\prime }-3d_{j}\cdot {\hat{r}}_{\mathrm{jj}}^{\prime }{\hat{r}}_{\mathrm{jj}}^{\prime }\cdot d_{j}^{\prime }}{r_{\mathrm{jj}}^{3}\prime }$, where $r_{\mathrm{jj}}^{\prime }$ is the distance between the emitters j and $j^{\prime }$, while ${\hat{r}}_{\mathrm{jj}}^{\prime }$ indicates the direction of a vector connecting them. This form of dipole–dipole coupling has been derived and applied in previous works focusing on electric dipole–dipole interactions in free-space [16] and their modifications near plasmonic nanostructures [17,19]. The same expression corresponds to the Förster resonance energy transfer (FRET) potential [39,40] where a pair of emitters is considered, one of them playing a role of a donor, the other, of an acceptor of a quantum of energy. This simple form is obtained by inserting the free-space Green’s function in Equation (7). It may be modified near plasmonic nanostructures influencing the form of the Green’s tensor, and as a result, modifying the range of dipole–dipole interactions/FRET [41,42], or due to the broad character of plasmonic resonances, its spectral characteristics. A particular example is related to plasmon—induced resonance energy transfer, in which energy transfer is enabled to acceptors whose transition line is centered at a frequency blue-shifted with respect to the donors [43].

Having mentioned the FRET, we need to explain how it could be possible to achieve a regime of irreversible energy transfer if the Hamiltonian in Equation (7) is Hermitian and describes reversible dynamics. Irreversibility arises naturally from introducing decoherence in the system. In FRET, the decoherence rate dominates over the multipole–multipole coupling ξ by 6 orders of magnitude, and is mostly related to nonradiative processes, e.g., collisions with molecules of a host medium [40]. In general, the shared photonic environment impacts the individual decay rates of the emitters through the famous Purcell effect [10,12], and may induce the corresponding collective rates which describe the sub- and superradiance phenomena in analogy to the Dicke model [16,19,44]. In most cases, decay and decoherence suppress the degree of stationary entanglement unless the subradiant channel is active through which highly entangled but weakly radiating states can be populated.

The individual rates are given by [36]

(10) γjj=2μ0ℏωj2∑mnDmr′†ωjDnrωjImGmnr′,r,ωj|r=rj,r′=rj,

and we account for the collective ones through the expression [36]

(11) γjj′=2πω¯2∑mnRmnω¯ImGmnr′,r,ω¯|r=rj,r′=rj′−Imnω¯ReGmnr′,r,ω¯|r=rj,r′=rj′,

with $j^{\prime }\neq j$. These rates enter the Liouville term that accounts for the non-Hamiltonian part of the dynamics of the density matrix ρ of the pair of quantum emitters

(12) $$L\left(\rho \right)=\sum_{j,j^{\prime }=1,2}D_{\gamma_{\mathrm{jj}}^{\prime }}\left(\rho ,\Sigma_{j},\Sigma_{j^{\prime }}^{†}\right),$$

where

(13) $$D_{\gamma }\left(\rho ,A,B\right)=\gamma \left(A\rho B-\frac{1}{2}BA\rho -\frac{1}{2}\rho BA\right).$$

With these tools at hand, one can evaluate the dynamics of the system through the Gorini–Kossakowski–Sudarshan–Lindblad equation [45,46]. However, we are interested to find its stationary solutions ρ which we deem more feasible for experimental investigations. For this purpose, we solve the stationary form of the equation

(14) $$-i\left[H,\rho \right]+L\left(\rho \right)=0.$$

Once the stationary density matrix is known, the degree of entanglement between the emitters can be evaluated e.g., in terms of concurrence [47]

(15) $$C\left(\rho \right)=\max\left\{0,\lambda_{1}-\lambda_{2}-\lambda_{3}-\lambda_{4}\right\},$$

where $\lambda_{i}$ stands for square roots of eigenvalues, in a descending order, of the matrix $\rho \tilde{\rho }$, and where $\tilde{\rho }=\left(\sigma_{y,1}⊗\sigma_{y,2}\right)\rho^{★}\left(\sigma_{y,1}⊗\sigma_{y,2}\right)$. Here, $\sigma_{y,j}=i\left(\sigma_{j}-\sigma_{j}^{†}\right)$.

### 2.3. Application

The following steps allow one to apply the theory introduced above to an arbitrary reasonable geometry.

- The Green’s tensor $G(r,r^{\prime },\omega )$ corresponding to the particular geometry under study should be found. Here, $r=r^{\prime }=r_{j}$ and $\omega =\omega_{j}$ for single-emitter effects, while $r=r_{j}$, $r^{\prime }=r_{j}^{\prime }$ and $\omega =\bar{\omega }$ for collective effects, are respectively positions of the probe and the source as well as their frequencies. For this purpose, we have used a freely-available MATLAB solver MNPBEM, as described in the Materials and Methods section;
- Relevant derivatives of the Green’s tensor should be evaluated according to the orientations of the multipolar moments (Equation (9)). The first derivatives correspond to the interference terms involving the electric-dipole component, while the second derivatives are related to the magnetic dipole and electric-quadrupole components. Description of higher-order components would require a generalization of the method;
- To evaluate the degree of stationary entanglement in terms of concurrence, one needs to insert the effective-Hamiltonian/Liouvillian parameters, calculated above, to Equation (14) for the stationary density matrix. The concurrence can be found directly according to the recipe in Equation (15). These derivatives scaled by the multipolar moments determine the emitter–emitter interaction strengths and decay rates, according to Formulas (7), (10) and (11).

We now apply this procedure to an example system. To acquire a fair estimation of different multipolar contributions to the degree of entanglement, we assume the dipole moments of each of the emitters to have lengths of 1 atomic unit and the following orientations: $d_{x}=1\;a.u.\approx 8.5\times 10^{-30}\;\mathrm{Cm}$, $m_{z}=1\;a.u\approx 1.9\times 10^{-23}\;{\mathrm{JT}}^{-1}$, and similarly for the electric-quadrupole moment of the transition $Q_{xy}=Q_{yx}=1\;a.u\approx 4.5\times 10^{-40}$${\mathrm{Cm}}^{2}$.

The nanoparticle is a silver nanosphere of 10 nm radius supporting a broad plasmonic resonance centered at a free-space wavelength of 360 nm, whose full-width at half maximum is of the order of 100 nm (not shown). The relative permittivity for silver at 360 nm is ϵ=−2.3020+0.26535i [48], the magnetic permeability is assumed to be equal to 1. As we show below, even for such a small nanoparticle, whose optical response is dominated by the electric-dipolar term, the contributions from higher multipoles to the interaction ξ, the decay rates $\gamma_{\mathrm{jj}}^{\prime }$, and to the stationary concurrence C are considerable. The system is illuminated with a *x*-polarized plane wave of frequency $\omega_{\mathrm{drive}}=5.2\times 10^{15}\;\mathrm{Hz}$ resonant with the nanosphere optical response. This drive is assumed resonant with the Lamb-shift-corrected transition frequency of Emitter 1 $\delta \omega_{1}=0$ and slightly detuned from the other emitter $\delta \omega_{2}=2$ GHz. Please note that this offset corresponds well to possible implementations, in which the Lamb shift correction depends on the emitter’s position with respect to the nanoparticle [23]. We fix the position of Emitter 1 at $r_{1}=\left(-13,0,0\right)$ nm with respect to the coordinate frame’s origin at the center of the sphere. We evaluate the resulting model parameters based on the electromagnetic Green’s tensor as described in detail in the subsections above. The parameters are calculated in dependence of position $r_{2}$ of the other emitter that is swept across the xy plane. The value of the coupling strength with the classical field for Emitter 1 is $\Omega_{1}=1$ GHz, while the couplings $\Omega_{2}$, ξ and decay rates $\gamma_{\mathrm{jj}}^{\prime }$ are position dependent. Please note that the values of the coupling to the classical field $\Omega_{j}$ only influence our final result: the concurrence, but not the vacuum-induced collective parameters ξ and $\gamma_{{\mathrm{jj}}^{\prime }}$.

Before we continue to discuss the results, it is important to comment on the case of free space, that is in the absence of the nanoparticle. In free space, the electric-dipole approximation works very well and the light–matter interaction is dominated by the electric-dipole channel. In consequence, the effective parameters, such as the spontaneous emission rate $\gamma_{\mathrm{jj}}$ due to the electric-dipole channel, overcome their analogons due the magnetic dipole and electric-quadrupole channels, respectively, by 5 and 6 orders of magnitude in the studied frequency range [36]. Similar scaling applies to $\gamma_{jj^{\prime }}$ and ξ. As we show below, this situation may be greatly modified near nanoparticles.

**Figure 1:**
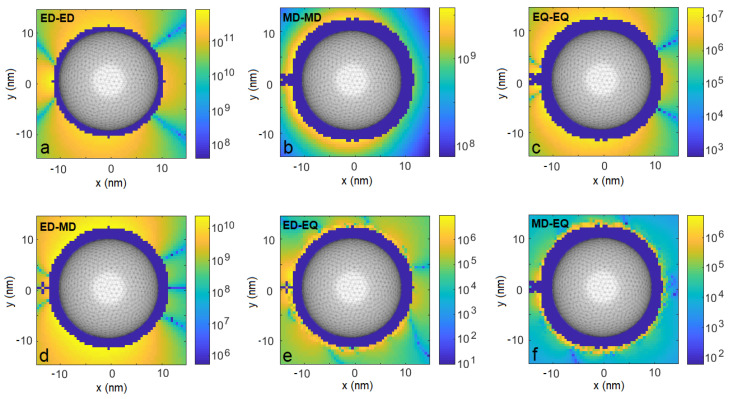
Coupling strengths |ξ| due to various light–matter interaction channels for Emitter 1 fixed at position $r_{1}=\left(-13,0,0\right)$ nm as a function of position $r_{2}$ of Emitter 2. Orientation of emitters’ multipolar moments described in the main text. (**a**). electric dipole–electric dipole coupling, (**b**). magnetic dipole–magnetic dipole coupling, (**c**). electric quadrupole–electric quadrupole coupling, (**d**). electric dipole–magnetic dipole coupling, (**e**). electric dipole–electric quadrupole coupling, (**f**). magnetic dipole–electric quadrupole coupling.

**Figure 2:**
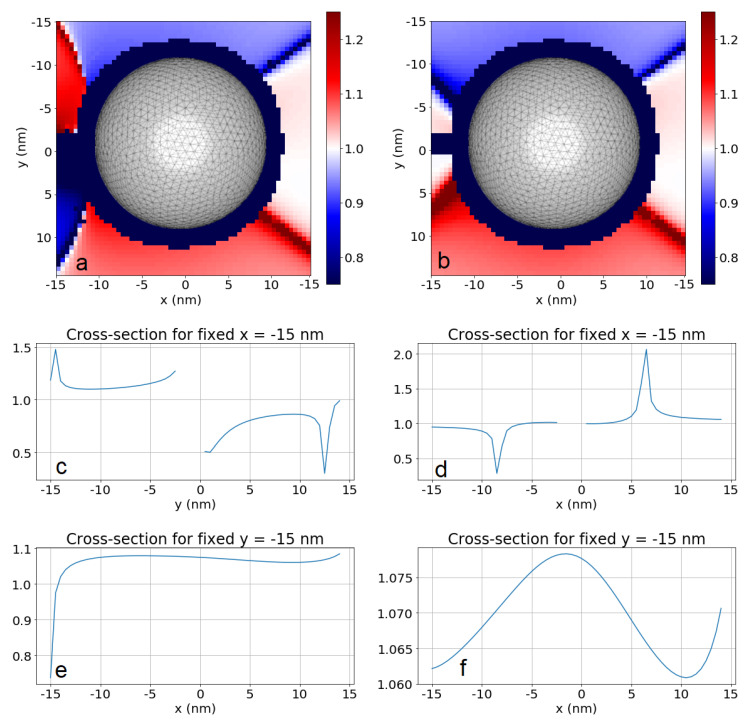
(**a**). Total coupling strength ξ compared to coupling strength $\xi_{ED-ED}$ from the electric-dipole channel only. (**b**). Total emission rate $\gamma_{12}$ compared to the emission rate $\gamma_{12,ED-ED}$ from the electric-dipole channel only. (**c**,**d**). Cross-sections of a,b for a fixed x=−15 nm. (**e**,**f**). Cross-sections of a,b for a fixed y=−15 nm.

In Figure 1, we compare the resulting coupling strengths |ξ| achieved due to the electric-dipole interaction channel (a), the magnetic-dipole channel (b), and the electric-quadrupole channel (c). The maps show |ξ| as functions of the position $r_{2}$. The navy-colored regions around the nanoparticle correspond to the positions less than 2 nm apart from the sphere, at which distance our results may not be reliable due to limitations of the software that was used to calculate the Green’s tensor [49] and therefore are not shown. We find that all the interaction channels are enhanced, but most importantly, their ratio with respect to the dominant channel is enhanced dramatically. The electric-dipole channel dominates by up to 2 (4) orders of magnitude near the nanoparticle over the magnetic-dipole (electric quadrupole) terms, as is evident from comparisons with Figure 1a–c. This means the relative strength of the higher-order multipoles (i.e., beyond the electric dipole) is enhanced with respect to the free-space case. Even more importantly, the impact of these channels is most visible through the interference terms (Figure 1d–f): The electric–magnetic dipole interference in Figure 1d is the main term responsible not only for the significant modulation of magnitude of the total coupling strength ξ with respect to the value obtained from the isolated electric-dipole channel, but also for the asymmetry with respect to the y=0 plane. We demonstrate this in Figure 2a. There, we find domains where the total interaction strength is robust with respect to positioning of Emitter 2 and modified by interference with the magnetic dipole by **±8%** for $r_{2}$ below and above the nanosphere, as it is shown in Figure 2e, up to even ±20% for the two emitters located on the same side with respect to the nanoparticle (Figure 2c). If the emitters are positioned on opposite sides, we find the influence of higher-order interaction channels to be limited to a few percent. Please note, however, that even in the latter case, the increase is substantial with respect to the free-space case. One can notice narrow spatial regions where the modulation may exceed 50% (Figure 2c for y=−14.0 nm and y=12.5 nm), but these extreme values are achieved in strongly limited volumes and the required experimental accuracy to exploit them would be very challenging to achieve.

We would like to elaborate on the antisymmetry of the result with respect to the y=0 plane. The physical origin of this effect lies in the interplay of electric- and magnetic-dipole moments of the emitters. The interference term arises as a result of the coupling of the magnetic field induced by the electric dipole corresponding to Emitter 1 and coupled to the magnetic dipole of Emitter 2, as well as the electric field induced by the magnetic dipole of Emitter 1 and coupled to the electric dipole of Emitter 2. The magnetic field induced by the electric dipole of Emitter 1 is dominated by the part scattered at the nanoparticle, which one can imagine as coming from a dipole induced at the nanoparticle. Naturally, the orientation of the magnetic field from such a dipole depends on the position of Emitter 2 which probes it, and in particular has opposite signs in the lower and upper halves of the xy plane.

**Figure 3:**
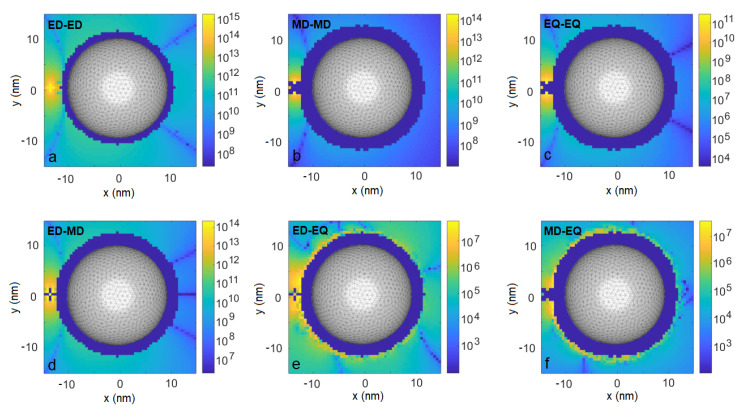
As in Figure 1, but for collective emission rates $|\gamma_{12}|$.

Similar effects can be identified on maps of the collective decay rates $\gamma_{12}$, whose absolute values are shown in Figure 3. These rates are rather robust with respect to respective positioning of the emitters: the rates due to the electric-dipole channel reach THz values in almost the entire simulation domain, while those originating from the magnetic-dipole and electric-quadrupole channel are of the order of tens of GHz and hundreds of MHz, respectively. All channels are enhanced for the emitters located very close to each other, i.e., less than a few nanometers apart. Again, the interference between the electric and the magnetic dipoles leads to a substantial modulation of the result (Figure 2d,f) by up to relatively stable values of ±8% in the lower and the upper halves of the xy plane, respectively, and even up to ±100% at specific spots (e.g., at x=−15 nm, y=6.5 nm).

**Figure 4:**
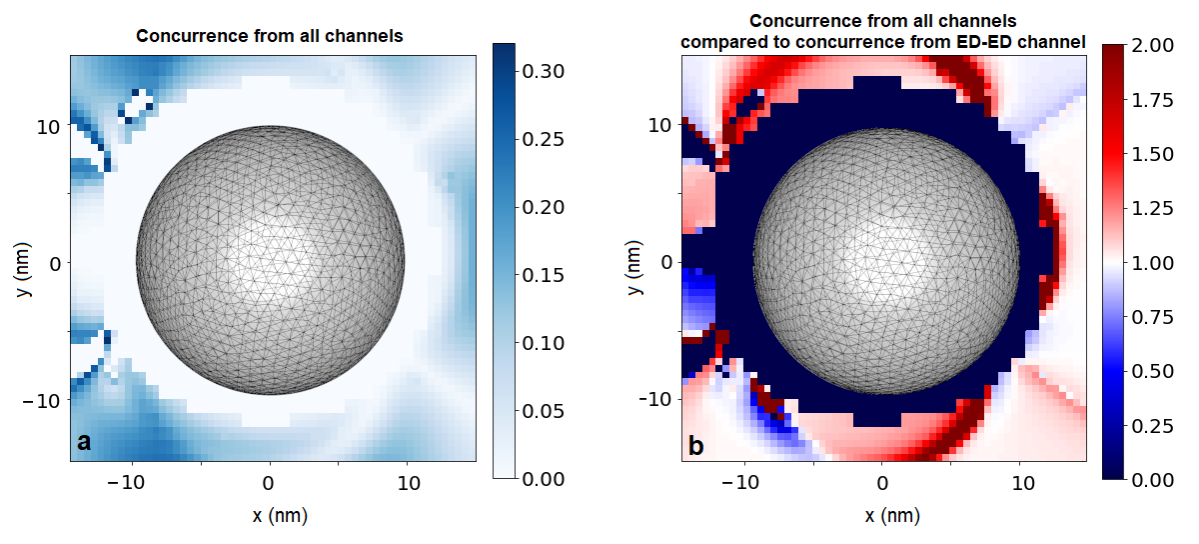
Concurrence due to all possible interaction channels (**a**) and the ratio of concurrence due to all possible channels compared with the concurrence due to electric-dipole channel only (**b**).

Both the multipole–multipole interaction carried by the photonic environment surrounding the nanostructure, and the collective decay rates induced by its presence, give rise to entanglement between the emitters, considerable even in the steady state. The degree of stationary entanglement of the emitters is shown in Figure 4 in terms of stationary concurrence C again in the function of the position of Emitter 2. Once more, the achieved values are robust against shifts in the emitter positioning: for almost all positions around the nanoparticle, C is close to its average value $C_{\mathrm{av}}=0.095$ (the averaging was performed over the investigated and reliable positions only and provides the order of magnitude for the effect). The spatial profile of the concurrence arising as a result of the interplay between all the investigated channels (Figure 4a) inherits the asymmetry that has been found in the coupling strengths and decay rates. As clearly visible from Figure 4b, the origin lies in the inclusion of higher-order interaction channels, in particular the interference between the electric- and magnetic-dipole channels, which influence the spatial profile of the result and modify it quantitatively by inducing the antisymmetry against the y=0 plane.

**Figure 5:**
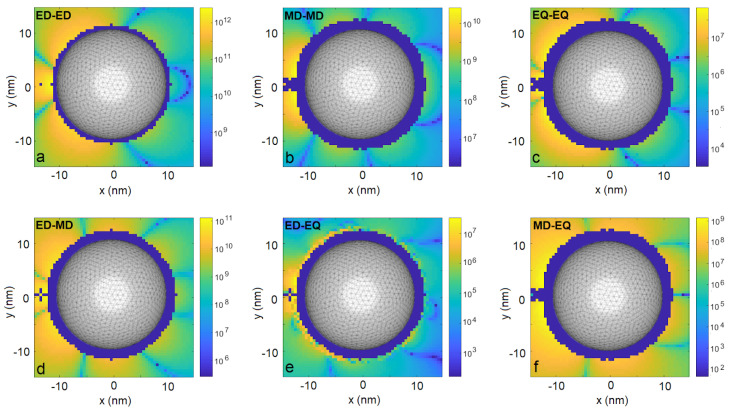
Coupling strengths |ξ| due to various light–matter interaction channels for an illumination wavelength of 350 nm, i.e., detuned from the plasmonic resonance. Emitter 1 is fixed at position $r_{1}=\left(-13,0,0\right)$ nm, coupling strengths are plotted as a function of position $r_{2}$ of Emitter 2. (**a**). electric dipole–electric dipole coupling, (**b**). magnetic dipole– magnetic dipole coupling, (**c**). electric quadrupole–electric quadrupole coupling, (**d**). electric dipole–magnetic dipole coupling, (**e**). electric dipole–electric quadrupole coupling, (**f**). magnetic dipole–electric quadrupole coupling.

To confirm the importance of the plasmonic resonance for the investigated effects, we repeat the calculations of the interaction strengths ξ and collective decay rates $\gamma_{\mathrm{jj}}^{\prime }$ for a drive at free-space wavelength of 350 nm, i.e., blue-shifted to a wing of the plasmonic resonance. The resulting emitter–emitter interactions are not transferred by the nanoparticle to its opposite side as efficiently as in the resonant case (compare Figure 1 and Figure 5). The weaker performance in the off-resonant case can be explained by less efficient field confinement and enhancement, affecting both values of fields and the derivatives, i.e., all considered interaction channels.

## 3. Discussion

Before we come to final conclusions, let us make several comments on the proposed scenario and the acquired results.

The scheme described above to induce multipole–multipole coupling between the emitters and eventually generate entanglement exploits a simple illumination scheme with a plane wave and does not require experimentally challenging preparation of the initial state of the quantum emitters. As demonstrated above, the resulting coupling strengths are rather stable across the simulation domain, which means precise positioning of the emitters is not required. Regarding the orientation of the multipolar moments of the emitters, a certain degree of control would however be beneficial. We have focused our analysis on the orientations in which higher-order interaction channels give rise to spatial asymmetry of the results. We deem this asymmetry to be a measure of the impact of these channels feasible for detection in experimental scenarios. Other orientations lead to similar orders of magnitude for enhancements, but may blur the asymmetry. As long as the dominant components of the transition moments are oriented as assumed above, all the qualitative features of our results should be conserved and significant.

Regarding the degree of entanglement, one could increase its stationary value through refined engineering of the nanoparticle, as well as through the application of a stronger driving field, which would help to overcome the decay rates. In the analysis above, we kept the drive moderate in order not to break the Markovian approximation [20], which is at the heart of the applied model [23].

We analyzed and discussed the influence of light–matter interaction channels beyond the electric dipole on collective phenomena that occur at the presence of a pair of quantum emitters near a silver plasmonic nanosphere. As a result, we found there to be significant influence on these quantities of the interference term between the electric and the magnetic dipoles, which modifies the results obtained within the typically applied electric-dipole approximation. This influence is of both a quantitative and qualitative character. First of all, the interference term may modify the achieved interaction strengths and decay rates by up to 8% in relatively stable spatial domains, and even by 100% at selected points. Secondly, it induces asymmetry of spatial distributions of the investigated quantities. On the other hand, our findings suggest that the influence of even higher-order terms would merely be a small correction, negligible in the particular case of geometry discussed in this work.

In this article, we described methods that could be exploited to include the electric-dipole, magnetic-dipole, and electric-quadrupole light–matter interaction channels in the analysis of quantum-optical scenarios involving small numbers of quantum emitters near nanoparticles. We confirmed the significance of terms beyond the electric-dipole approximation for multipole–multipole coupling, collective decay, and the degree of entanglement of emitters at close vicinity of a silver nanosphere. In the discussed example, we found the dominant correction to originate from the magnetic dipole–electric dipole interference channel. Other geometries where the electric-quadrupole channel may be enhanced were also proposed [50,51,52,53] and we expect them to have an impact on the collective effects considered here. Finally, nanostructures made of dielectrics or including dielectric components which support magnetic response at low absorption losses might be used for similar purposes [54,55,56,57,58].

## 4. Materials and Methods

Detailed introduction to the Green’s tensor’s formalism for field quantization in dispersive media can be found in [21]. Derivation of effective Hamiltonian parameters beyond the regime of electric-dipole approximation is given in [36].

Calculations of electromagnetic Green’s tensor around the investigated nanostructure were performed using the Metallic Nanoparticle Boundary Element Method (MNPBEM) toolbox for Matlab [49]. According to the developers of MNPBEM, to assure reliable results, one should calculate fields at a distance from the particle’s surface not smaller than the mean distance between the collocation points of the surface discretization, which in our example is equal to approximately 1 nm. To evaluate the impact of higher-order channels, first and second derivatives of the field are necessary. As a result, the reliable distance from the nanoparticle grows to 2 nm. Scripts are available from the corresponding author upon reasonable request. They can be directly generalized to account for other nanostructure geometries. Concurrence was calculated directly from formula (15) assuming the Markovian approximation holds.
